# Core outcome measures in perioperative and anaesthetic care

**DOI:** 10.1186/1745-6215-16-S1-P2

**Published:** 2015-05-29

**Authors:** Oliver Boney, Ramani Moonesinghe, Mike Grocott

**Affiliations:** 1 Surgical Outcomes Research Centre, University College Hospital, London, UK; 2 National Institute of Academic Anaesthesia, Royal College of Anaesthetists, London, UK

## Background

There is no current consensus within the anaesthetic and perioperative research community on what outcomes are important, nor how to measure them. This leads to heterogeneity of outcome reporting in randomised controlled trials (RCTs), and selective outcome reporting – i.e. a bias towards reporting only statistically significant outcomes [1]. Here we describe an initiative to develop a Core Outcome Set for Anaesthetic and Perioperative Research.

## Methods

Developing a Core Outcome Set will involve the following stages:

1. Identify and engage relevant stakeholders (patients, healthcare professionals, academic researchers)

2. Identify potential core outcomes: We are conducting systematic reviews of RCTs published from 2005 to 2014 in anaesthesia, surgery and perioperative medicine, to gain a broad overview of the range of outcomes measured and reported in the existing literature. Two systematic reviews will be conducted, one describing clinical outcomes, the other describing patient-reported outcomes. We will also seek views from patients and the public, and from healthcare professionals, on any additional outcomes that should be considered for inclusion in the core set.

3. Iterative Delphi methodology to achieve consensus regarding what outcomes should be included in the final Core Outcome Set

4. Publicise, promote and disseminate the Core Outcome Set for use in future anaesthetic and perioperative medicine research

## Discussion

The importance of robust outcome measurement in perioperative research cannot be overstated. A clear need thus exists for a standardised ‘core’ outcome set. This project aims to bring together patients, clinicians and researchers to analyse outcome measurement in perioperative care, and to agree a standardised core outcome set by mutual consensus.
